# Nanoparticle-Based Vaccines Against Respiratory Viruses

**DOI:** 10.3389/fimmu.2019.00022

**Published:** 2019-01-24

**Authors:** Soultan Al-Halifa, Laurie Gauthier, Dominic Arpin, Steve Bourgault, Denis Archambault

**Affiliations:** ^1^Département de Chimie, Université du Québec à Montréal, Montreal, QC, Canada; ^2^Quebec Network for Research on Protein Function, Engineering and Applications, PROTEO, Quebec, QC, Canada; ^3^Département des Sciences Biologiques, Université du Québec à Montréal, Montreal, QC, Canada; ^4^Faculté de Médecine Vétérinaire, Centre de Recherche en Infectiologie Porcine et Avicole (CRIPA), Université de Montréal, St-Hyacinthe, QC, Canada

**Keywords:** respiratory viruses, nanocarriers, nanovaccine, mucosal sites, immune response

## Abstract

The respiratory mucosa is the primary portal of entry for numerous viruses such as the respiratory syncytial virus, the influenza virus and the parainfluenza virus. These pathogens initially infect the upper respiratory tract and then reach the lower respiratory tract, leading to diseases. Vaccination is an affordable way to control the pathogenicity of viruses and constitutes the strategy of choice to fight against infections, including those leading to pulmonary diseases. Conventional vaccines based on live-attenuated pathogens present a risk of reversion to pathogenic virulence while inactivated pathogen vaccines often lead to a weak immune response. Subunit vaccines were developed to overcome these issues. However, these vaccines may suffer from a limited immunogenicity and, in most cases, the protection induced is only partial. A new generation of vaccines based on nanoparticles has shown great potential to address most of the limitations of conventional and subunit vaccines. This is due to recent advances in chemical and biological engineering, which allow the design of nanoparticles with a precise control over the size, shape, functionality and surface properties, leading to enhanced antigen presentation and strong immunogenicity. This short review provides an overview of the advantages associated with the use of nanoparticles as vaccine delivery platforms to immunize against respiratory viruses and highlights relevant examples demonstrating their potential as safe, effective and affordable vaccines.

## Introduction

Lower respiratory tract infections (LRTIs) constitute a major public health burden worldwide. LRTIs represent a leading cause of human mortality and morbidity, causing annually over 3 million deaths worldwide (1). Among these infections, about 80% of LRTI cases are caused by viruses (2). In most cases, these pathogens enter the host via airborne transmissions (e.g., droplets or aerosols), replicate efficiently in the respiratory tract and cause clinical manifestations, ranging from fever to bronchiolitis and pneumonia (3). In addition, LRTIs associated with viruses represent an important source of economic loss for livestock and poultry industry as these infections predispose animals to secondary bacterial infections (4–6).

Viruses infecting the human lower respiratory tract include the influenza virus, the respiratory syncytial virus (RSV), the parainfluenza virus and the adenovirus (7, 8). Seasonal influenza virus epidemics result in a significant burden of disease in children and elderlies and account for 3–5 million cases of severe illness and for nearly 290,000–650,000 deaths worldwide each year (9). RSV and parainfluenza virus infections are the leading cause of hospitalization for acute respiratory infections in young children, causing 45 and 40% of pediatric hospitalizations, respectively (10, 11). Adenovirus infections account for 3–5% of LRTIs cases in children and can be fatal for immunocompromised patients (12). In general, respiratory viruses represent a major health problem in infants, young children, immunocompromised patients and the elderly population. According to Global Burden of Diseases (GBD), 74% of deaths associated with LRTIs represent these vulnerable patient groups (13).

Vaccination remains the most cost-effective strategy to fight against infectious diseases. Conventionally, vaccine formulations consist of attenuated viruses, killed pathogens (inactivated) or subunit protein antigens, which elicit a specific immune response. These vaccine formulations have allowed the prevention, or the control, of several important diseases including rubella, yellow fever, polio and measles, and, in the case of smallpox, even eradication (14, 15). Considerable efforts have been devoted for the development of efficient vaccines against LRTIs, including inactivated/fragmented trivalent or quadrivalent seasonal vaccines against influenza type A and type B viruses such as Influvac® (16), Vaxigrip® (17), and Fluzone®(18) as well as live attenuated vaccines such as Nasovac® and Flumist® for nasal administration in young children (19, 20). Nevertheless, live-attenuated vaccines against influenza virus suffer from safety concerns due to their nature and represent a risk for elderly and immunosuppressed humans (21). Besides, killed pathogen vaccines and virus-derived subunit vaccines induce weaker immune responses and often require the use of an adjuvant to boost efficiency (22).

Several promising vaccines are currently evaluated in the clinics for different respiratory viruses (23). These new vaccine formulations aim to be safer and more efficient compared to traditional vaccines based on attenuated viruses, killed pathogens and subunits. Nevertheless, the high level of antigenic drift (genetic mutations) of some viruses, such as the influenza virus, reduces the efficacy of vaccines and needs to be addressed (24). Therefore, while improving safety and efficiency, vaccines should also be less sensitive to antigenic drift. The concept of “universal vaccine” is critical for viruses like the influenza virus, and new formulations to induce broad-spectrum immunity are being investigated. In the next sections, we discuss the advantages of using nanoparticle formulations against respiratory viruses and we highlight relevant examples of the use of nanoparticles as safe, effective, and affordable vaccines.

## Nanoparticles, an Alternative Approach to Conventional Vaccines

**Figure 1:**
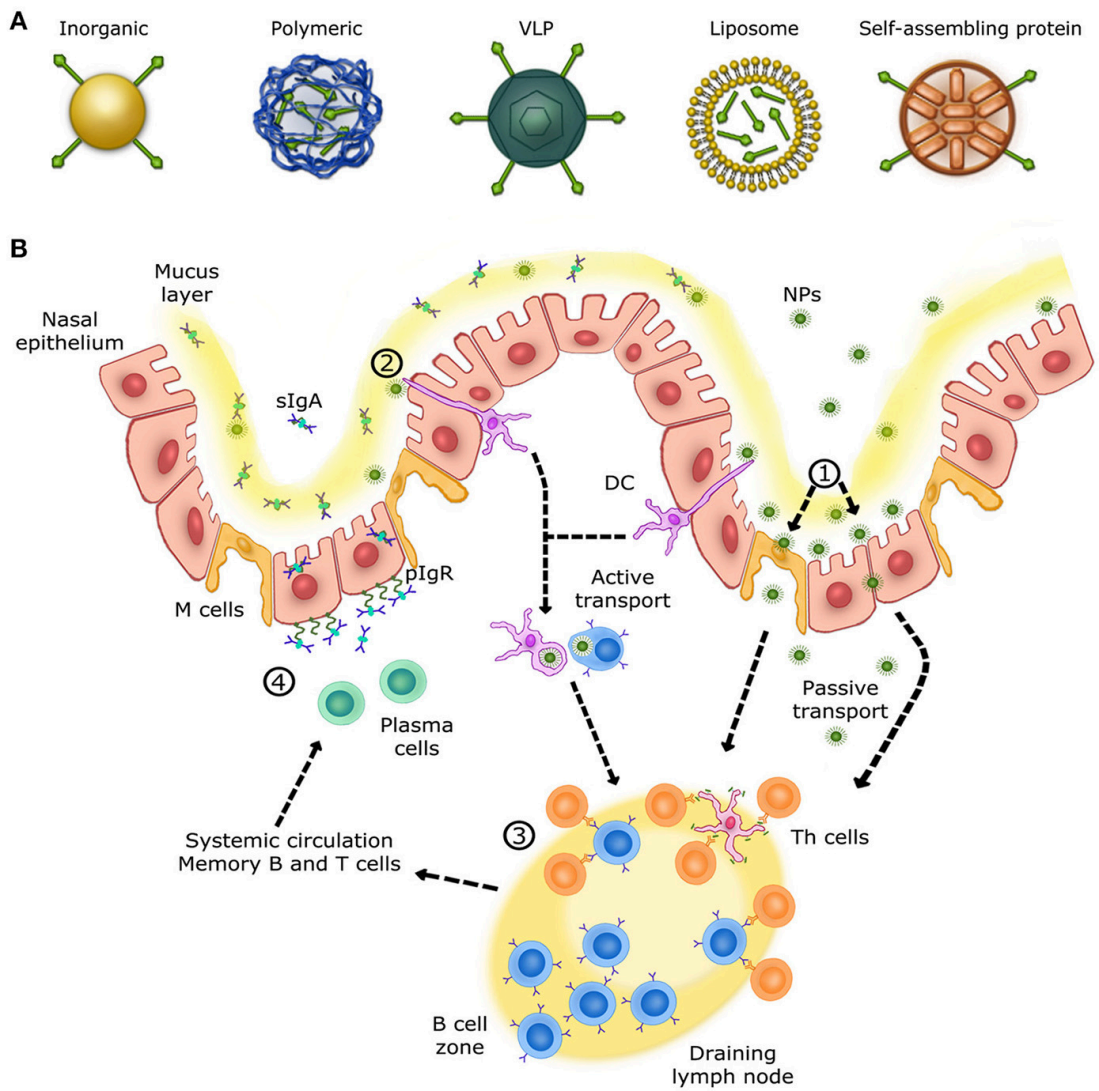
Overview of the immune response in the upper respiratory tract. **(A)** Schematic view of different nanoparticles used for intranasal vaccination. **(B)** Mechanisms of NALTs immune responses in the upper respiratory tract. (1) Nanoparticles are transcytosed from the mucus layer into the nasal epithelial tissues by micro-fold cells (M cells) or passively diffuse through epithelial cell junctions. (2) Other nanoparticles are captured and internalized by DCs (dendritic cells) from their extension through epithelial junctions and by other APCs, such as B cells. (3) Cells that have encountered nanoparticles migrate to the nearest lymph node in order to activate naive T helper cells. Once activated, T helper cells activate B cells that have encountered the same antigen presented by nanoparticles. Activated B cells proliferate in the lymph node (B cell zone) and, once mature, enter systemic circulation in order to reach the inflammation site. IgA+ B cells locally differentiate into antibody-secreting plasma cells to produce IgA dimers. (4) IgA dimers are secreted via polymeric Ig receptor (pIgR) at the mucosal surface. NALT immune response induces long-lasting memory B and T cells able to trigger a rapid recall response.

The use of particles as nanoplatforms displaying relevant antigenic moieties is appealing as an alternative approach to conventional vaccines. These nano-sized materials can be obtained from biological sources and/or can be synthetic. Currently, there is a large variety of particles evaluated as antigen carriers, including inorganic and polymeric nanoparticles, virus-like particles (VLPs), liposomes and self-assembled protein nanoparticles (Figure 1A). The advantages of these materials reside primarily in their size (at least one dimension should be at the nanometer level), since many biological systems such as viruses and proteins are nano-sized (25). Nanoparticles can be administered via sub-cutaneous and intramuscular injections, or can be delivered through the mucosal sites (oral and intranasal), and penetrate capillaries as well as mucosal surfaces (26, 27). Recent progresses have allowed the preparation of nanoparticles with unique physicochemical properties. For instance, size, shape, solubility, surface chemistry, and hydrophilicity can be tuned and controlled, which allows the preparation of nanoparticles with tailored biological properties (28). Moreover, nanoparticles can be designed to allow the incorporation of a wide range of molecules including antigens which makes them highly interesting in vaccinology (29, 30).

Incorporation of antigens in nanoparticles can be achieved by encapsulation (physical entrapment) or by conjugation (covalent functionalization) (21). Studies have demonstrated that nanoparticles could protect the native structure of antigens from proteolytic degradation and/or improve antigen delivery to antigen-presenting cells (APCs) (31). In addition, nanoparticles incorporating antigens can exert a local depot effect, ensuring prolonged antigen presentation to immune cells (32). Interestingly, nanoparticles have also shown intrinsic immunomodulatory activity (33). For instance, nanoparticles such as carbon nanotubes (CNTs), carbon black nanoparticles, poly(lactic-co-glycolic acid) (PLGA) and polystyrene nanoparticles, titanium dioxide (TiO_2_) nanoparticles, silicon dioxide (SiO_2_) nanoparticles, and aluminum oxyhydroxide nanoparticles have been reported to induce NLRP3-associated inflammasome activation (34). In fact, once internalized by APCs, these nanoparticles provide signals that trigger lysosomal destabilization and the production of reactive oxygen species (ROS), leading to the release of lysosomal contents, including the cysteine protease cathepsin B. This protease is sensed by NLRP3, which subsequently activates the formation of the inflammasome complex (35–39). Subsequently, interleukins are produced as downstream signaling events, leading to the recruitment and/or activation of immune cells (35, 40–45). Taken together, these properties advocate that nanoparticles are promising antigen carriers and immune cell activators for vaccination.

## Nanoparticles and the Respiratory Tract Immune System

The respiratory mucosa represents the primary site for invasion and infection by a virus whose replication occurs in the ciliated cells of the upper respiratory tract (URT). Subsequently, infection spreads to the low respiratory tract (LRT) by virus-rich secretions and by infected cell debris from the URT (46). Nasal-associated lymphoid tissue (NALT), the first site for inhaled antigen recognition located in the URT, is an important line of defense against respiratory viruses. NALT is present in rodents, birds and primates (47). This structure is characterized by aggregates of lymphoid cells located in the nasopharyngeal cavity (48). In human, the Waldever's ring, made of adenoid and tonsil, is considered as the equivalent of NALT structure, which contains various narrow epithelial channels. NALT comprises aggregates of lymphoid follicles (B-cell areas), interfollicular areas (T-cell areas), macrophages and dendritic cells (DCs) (Figure 1B), which, when activated, support the clearance of infectious agents (46, 48, 49). Accordingly, NALT is considered as an inductive site for humoral and cellular immune responses and represents a promising target for vaccines against respiratory viruses. Ideally, nanovaccines would follow a path similar to respiratory viruses in order to efficiently deliver antigens to NALT and trigger a specific mucosal immune response. Therefore, formulation, size and antigen exposition are critical aspects when designing nanovaccines targeting NALT. Most respiratory viruses have an average diameter size ranging between 20 and 200 nm (50–53). Thus, in addition of being safe and immunogenic on its own, a nanovaccine should have a size similar to viruses while incorporating relevant antigens (54).

Over the last decade, a number of nanoparticles have been designed to mimic respiratory viruses in terms of size, shape and surface property in order to target NALT as well as to raise humoral and cellular immune responses (21, 55, 56). First, beside a nanoparticle size of 20–200 nm in diameter to match the size of most respiratory viruses, nanoparticles should be preferably positively charged. In fact, positively charged polymeric, phospholipidic, metallic, inorganic, and protein-based nanoparticles have shown stronger immune responses compared to their negatively charged counterparts (21, 57). Second, the incorporation of antigens/epitopes within or on the surface of the nanoparticles can be challenging and requires advanced approaches in chemical and/or biological engineering (21). The most common strategy is to encapsulate or entrap antigens/epitopes within the nanoparticles. In this case, nanoparticles are used to protect the antigen/epitopes and deliver them to NALT (58–60). Nanoencapsulation can be achieved by using different procedures, including nanoprecipitation and oil in water (o/w) emulsion (61). Alternatively, antigens can be attached and exposed on the nanoparticle surface. This strategy aims at mimicking viruses. Conjugation of antigenic epitope can be performed directly on the nanoparticles using different chemical reactions like the disulfide bond and the thiolate-gold bond formation (62–64). Otherwise, it can be achieved by first preparing an epitope-functionalized self-assembling unit, which upon self-assembly form nanoparticles decorated with the antigen (65–67). Third, the formulation and administration strategies are also critical aspects to consider. Vaccines administered via subcutaneous or intramuscular injection induce systemic immunity and usually, a weak mucosal response is observed. On the other hand, mucosal vaccination, either oral or intranasal delivery, induces humoral, and cellular immune responses at the systemic level and the mucosal surfaces, which is more effective in the protection against respiratory viruses (68, 69). Studies have demonstrated that vaccination via the intranasal route provides a better protection when compared to subcutaneous immunization in the context of respiratory pathogens and mucosal immunity. Intranasal vaccination led to higher antigen-specific lymphocyte proliferation, cytokine production (interferon-γ, interleukins) and induction of antigen-specific IgA antibody (70–74). A promising formulation strategy is the intranasal spray, which delivers conveniently and safely the nanovaccines directly to the respiratory mucosa (75–77). However, the number of clinical trials using nanovaccine formulations for intranasal delivery, including spray dried nanovaccines, is limited. This is mostly associated with the difficulty of keeping the nanovaccine integrity during the entire formulation process (76). Moreover, the immune response is particularly sensitive to the nature, size, shape, and surface properties of the nanoparticles as well as to the density and the potency of the antigens. Thus, it is very challenging to predict the effect of a given nanovaccine on the immune system. In addition, nanoparticles have some limitations associated with their synthesis, or preparation, and their properties. These include limited antigen loading, low synthesis yield, poor targeting capability to immune cells, limited manufacturability, and, in some cases, toxicity (78–80). These drawbacks can lead to side effects and/or poor immunogenicity, which precludes their clinical usage. Besides, little is known about the interactions between nanoparticles and immune cells. In fact, their adjuvant effect and their ability to activate the immune system still remain unclear and need to be better understood at the molecular level (81). Nonetheless, nanoparticle formulations have recently revealed promising results against respiratory virus infections (Table 1) and relevant examples will now be discussed.

**Table 1:** Nanoparticle-based vaccines against respiratory viruses delivered via the intranasal route.

**Material**	**Size (nm)**	**Virus**	**Antigen/Epitope**	**Adjuvant**	**References**
**POLYMERIC NANOPARTICLES**					
PLGA	225.4	Bovine parainfluenza 3 virus (BPI3V)	BPI3V proteins	–	(82)
	200–300	Swine influenza virus (H1N2)	Inactivated virus H1N2 antigen	–	(83)
γ-PGA^a^	100–200	Influenza (H1N1)	Hemagglutinin	–	(84)
Chitosan	140	Influenza (H1N1)	H1N1 antigen	–	(85)
	300–350	Influenza (H1N1)	HA-Split	–	(86)
	571.7	Swine influenza virus (H1N2)	Killed swine influenza antigen	–	(87)
	200–250	Influenza (H1N1)	M2e	Heat shock protein 70c	(88)
HPMA/NIPAM	12–25	RSV	F protein	TLR-7/8 agonist	(89, 90)
Polyanhydride	200–800	RSV	F and G glycoproteins	–	(91, 92)
**SELF-ASSEMBLING PROTEINS AND PEPTIDE-BASED NANOPARTICLES**					
N nucleocapside protein of RSV	15	RSV	RSV phosphoprotein	R192G	(93)
	15	RSV	FsII	Montanide™ Gel 01	(94)
	15	Influenza (H1N1)	M2e	Montanide™ Gel 01	(95)
Ferritin	12.5	Influenza (H1N1)	M2e	–	(96)
Q11	–	Influenza (H1N1)	Acid polymerase	–	(97)
**INORGANIC NANOPARTICLES**					
Gold	12	Influenza	M2e	CpG	(64)
**OTHERS**					
VLP	80–120	Influenza (H1N1)	Hemagglutinin	–	(98)
	80–120	Influenza (H1N1, H3N2, H5N1)	M2e	–	(99)
	80–120	RSV	F protein et G glycoprotein of RSV and M1 protein of Influenza	–	(100)
ISCOM^b^	40	Influenza (H1N1)	Hemagglutinin	ISCOMATRIX	(101, 102)
DLPC liposomes^c^	30–100	Influenza (H1N1)	M2, HA, NP	MPL and trehalose 6,6′ dimycolate	(103)

a *Poly-γ-glutamic acid*.

b *Quillaia saponin, cholesterol, phospholipid, and associated antigen*.

c *Dilauroylphosphatidylcholine*.

## Polymeric Nanoparticles

A polymer consists of a large molecule constructed from monomeric units. Depending on the construction, polymers can be linear, slightly branched or hyperbranched (3D network) (104). Polymeric nanoparticles can be either obtained from the polymerization of monomeric units or from preformed polymers. These nanoparticles are attractive in the medical field due to their adjustable properties (size, composition, and surface properties), which allow controlled release, ability to combine both therapy and imaging (theranostics), and protection of drug molecules (105–107). For example, poly(lactic-co-glycolic acid) (PLGA) is a biodegradable and biocompatible polymer approved by the Food and Drug Administration (FDA) and European Medicines Agency (EMA) for use in humans. This is due to its ability to undergo hydrolysis *in vivo*, resulting in lactic acid and glycolic acid metabolites, which are efficiently processed by the body (108). PLGA can be engineered to form nanoparticles capable of encapsulating different types of biomolecules and release them sustainably over time (108–111). These nanoparticles can encapsulate antigens and prevent their degradation over 4 weeks under physiological conditions, which is critical for mucosal vaccination (112). Moreover, PLGA-NPs promote antigen internalization by APCs and facilitate antigen processing and presentation to naïve lymphocytes (113, 114). For instance, spherical PLGA-NPs (200–300 nm of diameter) were used to encapsulate an inactivated Swine influenza virus (SwIV) H1N2 antigens (KAg) via water/oil/water double emulsion solvent evaporation (83). It was observed that pigs vaccinated twice with this preparation and challenged with a virulent heterologous influenza virus strain, have a significantly milder disease in comparison to non-vaccinated animals. This observation correlated closely with the reduced lung pathology and the substantial clearance of the virus from the animal lungs. Other polymeric nanoparticles, such as chitosan, a natural polymer composed of randomly distributed β-(1–4)-linked d-glucosamine and N-acetyl-d-glucosamine, and N-(2-hydroxypropyl)methacrylamide/N-isopropylacrylamide (HPMA/NIPAM), were also investigated as intranasal vaccines against respiratory viruses (85–90, 115–121). Overall, polymeric nanoparticles have many advantages, including biocompatibility (122), antigen encapsulation and stabilization (123, 124), controlled release of antigens and intracellular persistence in APCs (125, 126), pathogen-like characteristics, and suitability for intranasal administration (126, 127). Nevertheless, the effect of the polymer properties (core chemistry, size, shape, surface properties) on the transport within the URT remains unknown. More studies are needed to better understand the effect of changing nanoparticle properties on their biological activities and to, ultimately, predict the fate of these nanocarriers upon their intranasal administration.

## Self-Assembling Protein Nanoparticles and VLPs

Self-assembling protein nanoparticles (SAPNs) are structures obtained from the oligomerization of monomeric proteins. The protein building blocks are mostly obtained through recombinant technologies and are considered safe for biomedical applications (128). SAPNs can be engineered to have a diameter ranging from 20 to 100 nm, similar to the sizes of many viruses and therefore, are considered as nanovaccine candidates against viruses, including respiratory viruses (128, 129). For example, SANPs, designed to elicit an immune response against RSV, have been explored using the nucleoprotein (N) from the virus nucleocapsid. The N protein is a major target of antigen-specific cytotoxic T-cell response. The self-assembly of N protein protomers led to the formation of supramolecular nanorings of 15 nm diameter (93). This platform was modified by fusing the FsII epitope targeted by monoclonal neutralizing antibody (palivizumab) to the N-protein, in order to form chimeric nanorings with enhanced immune response and virus protection against RSV. The results showed reduced virus load in the lungs of challenged mice (94). Similarly, chimeric nanorings displaying 3 repeats of the highly conserved ectodomain of the influenza virus A matrix protein 2 (M2e), were prepared by recombinant technologies (95). When administrated via the intranasal route, these M2e-functionalized nanorings induced local production of mucosal antibodies and led to mice protection (95). These N-nanorings are interesting for intranasal delivery of antigen due to their similarities with respiratory viruses in term of size and structure (sub-nucleocapsid-like superstructures). Other examples of SAPNs as potential nanovaccines against respiratory viruses include the capsid protein of the papaya mosaic virus (PapMV), the purified coronavirus spike protein and ferritin, which are self-assembling proteins that form rod-shaped and nearly spherical nanostructures, respectively (96, 130–140). Recently, assemblies composed of four tandem copies of M2e and headless HA proteins were prepared and stabilized by sulfosuccinimidyl propionate crosslinking, showing the possibility of generating protein nanoparticles almost entirely composed of the antigens of interest (141).

VLPs are spherical supramolecular assemblies of 20–200 nm diameter, which result from the self-assembly of viral capsid proteins. These particles are free from genetic materials and have the advantage of mimicking perfectly the structure and the antigenic epitopes of their corresponding native viruses. Therefore, this repetitive antigen display promotes efficient phagocytosis by APCs and subsequent activation (142–146). Recently, Lee and colleagues demonstrated that intranasal delivery of influenza-derived VLPs expressed in insect cells and exposing 5 repeats of the M2e epitopes, confers cross protection against different serotypes of influenza viruses by inducing humoral and cellular immune responses (99).

SAPNs and VLPs are thus attractive but their formulation into stable and spray dried vaccines for intranasal injection can be challenging and may require the use of surfactants and saccharides (147). In the last decades, self-assembling peptides (SAPs) have also been investigated as intranasal nanovaccines against respiratory viruses due to their straightforward chemical synthesis and their storage stability upon lyophilization (97).

## Inorganic Nanoparticles

There are many inorganic nanoparticles suitable for biomedical applications, including superparamagnetic nanoparticles (iron oxide nanoparticles), quantum dots and plasmonic nanoparticles (gold and silver nanoparticles). Inorganic materials are mostly used as tools with improved therapeutic efficacy, biodistribution and pharmacokinetics. However, inherently, plain inorganic core nanoparticles would not be suitable in biological fluids due to particle aggregation. Therefore, in the medical field, these nanoparticles are often coated with organic molecules via adsorption or chemical reactions. In fact, these biocompatible nanoparticles can be described as complex hybrids materials with an inorganic core and an organic outer shell (148, 149). Among inorganic nanoparticles, the most commonly used for vaccination are gold nanoparticles (AuNPs). AuNPs are readily internalized by macrophages and dendritic cells, and induce their activation (150, 151). Large scale production is possible with strict control on particle size and ease of functionalization using the strong affinity between thiol groups and gold. Thiol groups can be attached to AuNP surface by forming thiolate–Au bonds (152–155). Furthermore, no immune response is elicited toward inert carriers like AuNPs (156). Thus, these nanoparticles are an appealing platform for nanovaccine engineering *via* antigen functionalization.

A wide range of molecules, including adjuvants and antigens can be conjugated on AuNPs at high density, resulting in improved immunogenicity and antigen presentation (157, 158). AuNPs can be formulated for intranasal administration and can diffuse into the lymph nodes, triggering robust antigen-specific cytotoxic T-cell immune responses (159, 160). Tao and coworkers have demonstrated that the peptide consensus M2e of influenza A viruses with a non-native cysteine residue at the C-terminal end could be attached on the AuNPs via thiolate–Au chemistry. The resulting M2e-AuNPs was administered by the intranasal route to mice with CpG (cytosine-guanine rich oligonucleotide) adjuvant, triggering a fully protective immune response against the influenza virus PR8 strain (161). More recently, it was demonstrated that this formulation could induce lung B cell activation and robust serum anti-M2e IgG response, with stimulation of both IgG1 and IgG2a subclasses (161). Additionally, this vaccination strategy led to protection against infection by the pandemic influenza virus strain, A/California/04/2009 (H1N1pdm) pandemic strain, influenza virus A/Victoria/3/75 (H3N2) strain and the highly pathogenic avian influenza virus A/Vietnam/1203/2004 (H5N1) (64). Although gold nanoparticles constitute an attractive platform for antigen conjugation, they can accumulate in organs such as liver and spleen for a long period, which could be ultimately associated with toxicity (162). Coating with biocompatible materials reduces their toxicity, although it can lead to alterations of the physicochemical and biological properties. Therefore, safety issues of AuNPs still need to be addressed.

## Conclusion and Perspectives

Engineered nanoparticles have demonstrated their potential as vaccine delivery platforms. They can be envisaged as both antigen nanocarriers and adjuvants. In all cases, intranasal administration of nanovaccines allows a convenient and safe delivery of the antigen to NALT, inducing mucosal and systemic immunity. Nonetheless, additional studies are still needed before their clinical translation. While intranasal vaccination of nanoparticles generates specific IgA antibody in the URT and leads to high survival rates in animal models, there are still limited studies on non-human primates, thus making nanoparticle's fate difficult to predict in a human URT. In addition, nanoparticle vaccines are generally functionalized with specific antigen(s), which result in an immune response targeted against these antigenic determinants. Considering antigenic drifts, the growing human population that needs to be vaccinated and the different type of viruses, the cost to address all these aspects would be too prohibitive to produce affordable vaccines. Consequently, the development of broad spectrum vaccines constitutes a critical need and we consider that nanovaccine engineering will contribute to achieve this objective.

## Author Contributions

SA-H, LG, DoA, SB, and DeA have participated in writing and preparation of the manuscript, and approved it for publication.

### Conflict of Interest Statement

The authors declare that the research was conducted in the absence of any commercial or financial relationships that could be construed as a potential conflict of interest.
